# Observation of others’ actions during limb immobilization prevents the subsequent decay of motor performance

**DOI:** 10.1073/pnas.2025979118

**Published:** 2021-11-15

**Authors:** Doriana De Marco, Emilia Scalona, Maria Chiara Bazzini, Arturo Nuara, Elisa Taglione, Nicola Francesco Lopomo, Giacomo Rizzolatti, Maddalena Fabbri-Destro, Pietro Avanzini

**Affiliations:** ^a^Istituto di Neuroscienze, Consiglio Nazionale delle Ricerche, Parma 43125, Italy;; ^b^Dipartimento di Medicina e Chirurgia, Università degli Studi di Parma, Parma 43125, Italy;; ^c^Centro di Riabilitazione Motoria di Volterra, Istituto Nazionale per l'Assicurazione contro gli Infortuni sul Lavoro (INAIL), Volterra 56048, Italy;; ^d^Dipartimento di Ingegneria dell'Informazione, Università degli Studi di Brescia, Brescia 25121, Italy;; ^e^Humanitas Clinical and Research Center – IRCCS, Rozzano 20089, Italy

**Keywords:** action observation, mirror mechanism, motor rehabilitation, early treatment

## Abstract

There is rich clinical evidence that observing normally executed actions promotes the recovery of the corresponding action execution in patients with motor deficits. In this study, we assessed the ability of action observation to prevent the decay of healthy individuals’ motor abilities following upper-limb immobilization. To this end, upper-limb kinematics was recorded in healthy participants while they performed three reach-to-grasp movements before immobilization and the same movements after 16 h of immobilization. The participants were subdivided into two groups; the experimental group observed, during the immobilization, the same reach-to-grasp movements they had performed before immobilization, whereas the control group observed natural scenarios. After bandage removal, motor impairment in performing reach-to-grasp movements was milder in the experimental group. These findings support the hypothesis that action observation, via the mirror mechanism, plays a protective role against the decline of motor performance induced by limb nonuse. From this perspective, action observation therapy is a promising tool for anticipating rehabilitation onset in clinical conditions involving limb nonuse, thus reducing the burden of further rehabilitation.

## Significance

In several clinical conditions, especially those related to orthopedic trauma or specific injuries of the peripheral nervous system, patients may experience a period of limb nonuse that has detrimental cascade effects on corticomotor organization and ultimately, on motor performance. During limb nonuse, treatments based on action observation may be suitable for stimulating the motor system via the mirror mechanism. Using short-term immobilization in healthy volunteers, our study showed that administering action observation during immobilization limits the movement alterations induced by limb nonuse. Given action observation’s protective role against the decline of motor performance, it represents a valid tool for early interventions during limb nonuse, thus reducing the burden of further motor rehabilitation.

There is rich clinical evidence that observing normally executed actions promotes the recovery of the corresponding action execution in patients with motor deficits. This procedure, which is based on the activation of the motor system via the mirror mechanism (1, 2), is called action observation treatment (AOT) (3, 4). The effectiveness of AOT in motor recovery has been demonstrated in several clinical conditions, including stroke (5–7), Parkinson's disease (8–10), multiple sclerosis (11), and cerebral palsy (12–16), as well as in patients with orthopedic trauma and postsurgical patients (17–19).

In recent decades, researchers have advanced covert motor approaches based on action observation other than AOT. Such approaches include mirror therapy (20, 21), which improves the symptoms resulting from absent or altered feedback from the affected side of the body [e.g., phantom pain in arm amputees (22, 23)] and may also enhance motor function in poststroke patients (24–26). More recently, noninvasive brain stimulation techniques, such as transcranial magnetic stimulation, transcranial direct current stimulation, and peripheral electrical stimulation, have been used to enhance motor recovery in neurological (27–29) and orthopedic patients (30). When tested in combination with interventions based on action observation, these approaches exhibited the ability to enhance the magnitude of treatment effects (31, 32).

In some of the abovementioned clinical conditions, especially those involving orthopedic trauma or affecting the peripheral nervous system, the patient may experience a period of limb nonuse. It has been demonstrated that limb nonuse (or disuse) induces a reduction in the size and excitability of the cortical representation of the immobilized limb, gradually leading to maladaptive plasticity changes and the appearance of motor alterations (33–35), which can interfere with the rehabilitative outcome. In this context, action observation is an effective treatment alternative when physical therapy is not applicable. The aim of the present study is to determine whether administering AOT during the immobilization period can limit the progressive impoverishment of motor performance—an effect researchers have yet to be establish.

To this end, a short-term immobilization (STI) was administered to healthy volunteers. This procedure is commonly used to model the neurophysiological changes leading to motor impairments in injured people (reviews are in refs. 36 and 37) because it minimizes the impact of confounding variables (e.g., immobilization duration, cause of immobilization, associated pain, potential comorbidities) and consequently, isolates the hypoactivity-induced effects on neurophysiological processes. Moreover, the use of healthy volunteers enables a within-subjects comparison between pre- and postimmobilization performance, whereas the use of clinical populations makes the same procedure virtually impossible due to the sudden nature of the injury.

The participants were subdivided into two groups: those receiving AOT and those receiving control stimulations. In both groups, the upper-limb kinematics of goal-directed movements was tested before and after the immobilization. We evaluated whether the motor performance of subjects who underwent AOT was better preserved after the immobilization compared with that of the control (CTRL) group, and we determined which aspects of movement organization were mostly affected by AOT.

The present study’s results could lead to the use of AOT during immobilization in a spectrum of clinical conditions in which the patient's movement is transiently impeded, thus favoring an early treatment onset and potentially limiting the extent of motor deficits to be later rehabilitated.

## Results

**Fig. 1.**
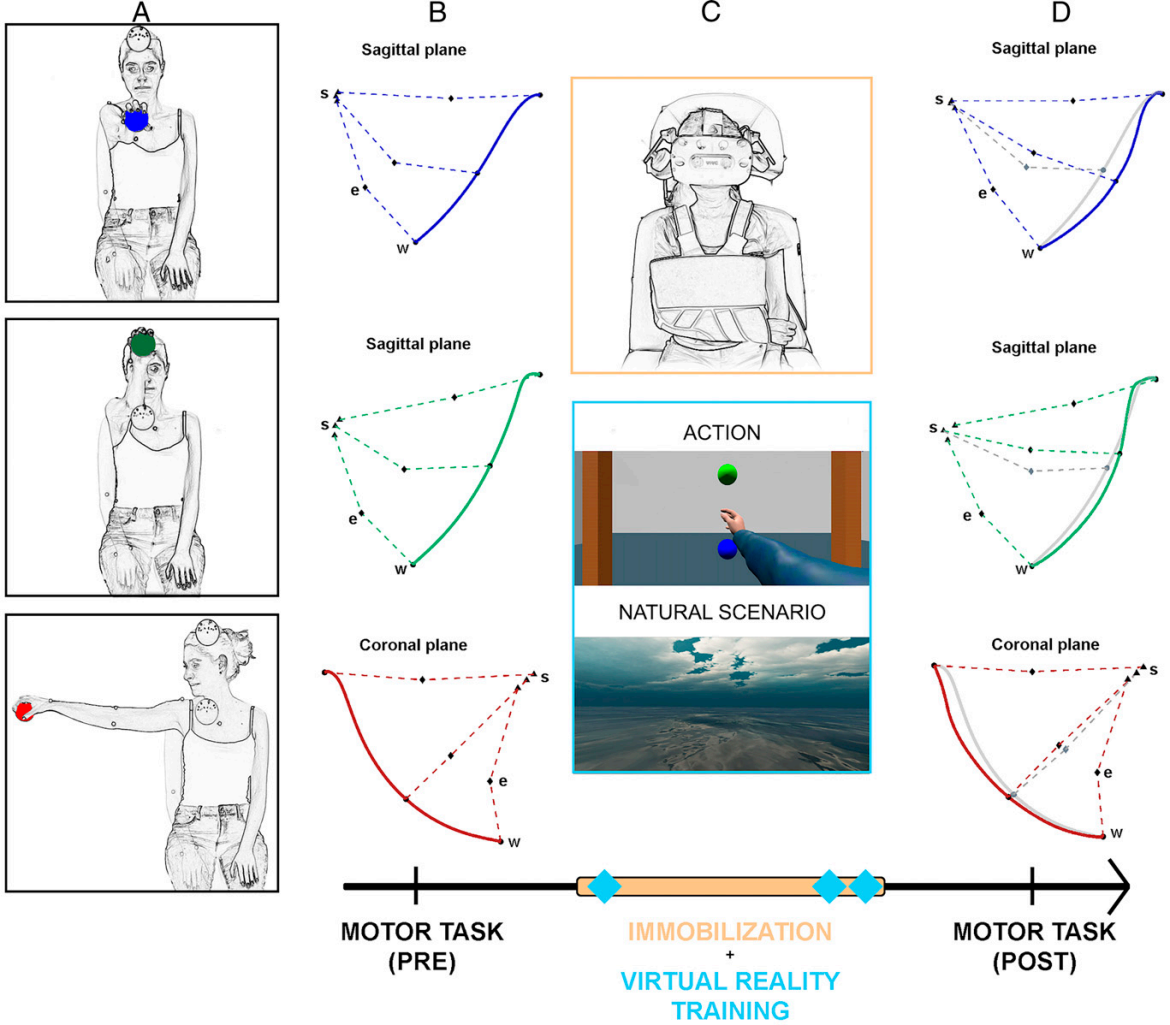
Experimental protocol. The figure must be read left to right, following the time line. (*A*) The actions executed by participants during the motor task (*Top*, A-Low; *Middle*, A-High; and *Bottom*, L-Low). (*B*) Example of wrist trajectories (solid lines) acquired during the execution of the three movements in their anatomical reference planes during the preimmobilization phase. The dashed lines connect shoulder (s), elbow (e), and wrist (w) joint centers captured at the beginning of the reaching motion, at maximum elbow flexion, and at the end of the reaching motion. (*C*) The immobilization period (orange bar) and VR sessions (blue diamonds) based on actions or natural scenarios for the AOT and CTRL groups, respectively. (*D*) Postimmobilization kinematics (wrist trajectories and joint center positions) for the same subjects shown in *B* depicted alongside the preimmobilization trajectory (gray lines). Note the lower postimmobilization elbow flexion, leading to a less ballistic wrist trajectory.

In order to assess AOT’s protective role against the motor impairments that typically occur after immobilization, we recorded the upper-limb kinematics of a group of healthy volunteers before and after arm immobilization (16 h). The participants were asked to perform three reach-to-grasp movements, which were distinguished by the location of the target object, as follows: 1) located anteriorly at the height of the subject’s shoulders (A-Low), 2) located anteriorly at the height of the subject’s head (A-High), and 3) located laterally at the height of the subject’s shoulders (L-Low) (Fig. 1*A*). During immobilization, half of the participants (the AOT group) repeatedly observed and imagined the same movements previously executed, whereas the other half (the CTRL group) observed natural scenarios for an equivalent amount of time.

No significant differences between AOT and CTRL groups at baseline were found for reaching duration (RD), reaching velocity peak (VP), or movement fractionation (MFr; all *P* > 0.18) (*SI Appendix*, Table S1). Table 1 reports the results for RD, VP, and MFr for the three tested movements. According to the ANOVA, all the variables showed a significant main effect of TIME. The worst impairments were observed immediately after bandage removal, namely at the first postimmobilization trial (T1), and all the subjects underwent a noticeable recovery over the course of the postimmobilization trials (from T1 to T10). This finding indicates that 16 h of immobilization was sufficient to induce a subtle but quantifiable alteration of kinematic performance, the recovery of which could be evaluated during the postimmobilization procedures.

**Table 1.** Differences among postimmobilization scores at T1, T4, and T9 relative to the average scores of preimmobilization for RD, VP, and MFr

Movement, index, and group	T1	T4	T9
A-Low			
RD (s)			
AOT	0.23 (0.05)	0.03 (0.03)	0.04 (0.03)
CTRL	0.33 (0.10)	0.09 (0.05)	0.00 (0.04)
VP (m/s)			
AOT	−0.22 (0.03)	−0.03 (0.03)	−0.03 (0.04)
CTRL	−0.29 (0.04)	−0.07 (0.04)	0.01 (0.04)
MFr (%)			
AOT	6.02 (3.54)	−0.79 (2.43)	−1.76 (2.45)
CTRL	10.44 (3.45)	5.62 (1.48)	5.47 (1.78)
A-High			
RD (s)			
AOT	0.16 (0.04)	0.08 (0.05)	0.03 (0.03)
CTRL	0.30 (0.08)	0.15 (0.06)	0.04 (0.02)
VP (m/s)			
AOT	−0.18 (0.05)	−0.03 (0.06)	−0.09 (0.03)
CTRL	−0.26 (0.07)	−0.17 (0.05)	−0.06 (0.04)
MFr (%)			
AOT	2.83 (1.72)	−1.77 (2.33)	−0.01 (1.51)
CTRL	9.12 (2.25)	4.88 (2.01)	3.69 (1.94)
L-Low			
RD (s)			
AOT	0.19 (0.05)	0.09 (0.05)	0.04 (0.05)
CTRL	0.14 (0.06)	0.03 (0.10)	0.00 (0.05)
VP (m/s)			
AOT	−0.15 (0.04)	−0.04 (0.05)	−0.07 (0.07)
CTRL	−0.16 (0.05)	−0.07 (0.06)	−0.02 (0.04)
MFr (%)			
AOT	6.11 (2.93)	3.03 (2.52)	2.88 (2.97)
CTRL	18.10 (3.32)	11.17 (3.42)	10.33 (3.31)

In each cell, mean differences and SEs are shown for each group (AOT and CTRL) and movement (A-Low, A-High, and L-Low).

The most interesting finding indeed concerns the effect of the GROUP factor, which appeared to be limited to MFr, a parameter indexing the relative ratio between the range of motion (ROM) for the elbow- and shoulder-joint angles of interest. Comparing pre- and postimmobilization scores, MFr scores were higher for CTRL participants relative to the AOT group for all the three movements [*F*(1, 38) = 7.55, *P* = 0.009, partial η^2^ = 0.16, Bayes Factor (BF)_10_ = 5.15]. A significant main effect of MOVEMENT also emerged, likely reflecting a stronger effect of the immobilization procedure on the coordination of shoulder and elbow joints in the L-low movement. However, no interaction effects were found, evidencing a similar effect of AOT on the three movement patterns. More detailed statistical analysis results for RD, VP, and MFr are reported in *SI Appendix*, Table S2.

As the MFr depends on the shoulder- and elbow-joint angles, we assessed the effects of immobilization (TIME factor) and AOT (GROUP factor) on each joint separately. The comparison between each postimmobilization trial and the average preimmobilization kinematics was performed using the linear fit method [LFM (38)], which returns three indexes (amplitude modulation [AM], *R*^2^, amplitude offset between the curves [AOff]) describing different features of the movement pattern. Considering that MFr scores vary according to elbow and shoulder ROM, we focused on the AM parameter, which indicates whether postimmobilization movements are more/less scaled in amplitude (AM > 1 and AM < 1, respectively) relative to baseline (T0). On the contrary, we did not expect variation in the *R*^2^ and AOff indexes, which test differences in the time course and offset of the joint angle curves, respectively.

**Fig. 2.**
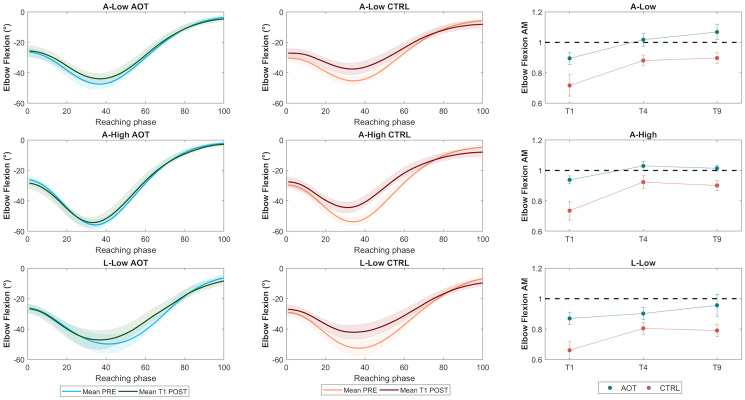
(*Left* and *Center*) Time courses of elbow angles averaged across preimmobilization trials (light colors) for the AOT and CTRL groups (green and red, respectively) for the three movements (A-Low, A-High, and L-Low); time courses of mean elbow angles acquired during the first trial of the postimmobilization phase (T1 post) are superimposed in corresponding dark colors. (*Right*) Means and SEs of AM evaluated at T1, T4, and T9 for the AOT and CTRL groups (green and red, respectively).

Fig. 2 depicts the average time course of the elbow flexion–extension angle in the preimmobilization phase and the first trial of the postimmobilization phase for the AOT and CTRL groups. The results showed that immobilization reduces the elbow ROM, in particular for the CTRL group. In support of this point, the ANOVA for AM (Fig. 2, *Right*) showed a significant main effect of GROUP [*F*(1, 38) = 10.98, *P* = 0.002, partial η^2^ = 0.22, BF_10_ =15.94], with CTRL participants systematically showing lower AM scores: that is, a higher level of joint stiffness for all movements relative to the AOT group.

A main effect of TIME was found [*F*(2, 76) = 10.06, *P* < 0.01, partial η^2^ = 0.21, BF_10_ > 100]. Post hoc comparisons revealed that T1 had systematically lower values than T4 and T9 (all *P* < 0.05) (*SI Appendix*, Table S2), underlining that the initial shrinkage of elbow movement was reversed over the course of the postimmobilization training. The absence of a significant GROUP × TIME interaction suggested that the recovery dynamics, present for all movements as indicated by the significant TIME effect, were not impacted by AOT. Rather, AOT seemed to preserve participants’ original motor abilities, reflected in a lower degree of impairment at T1 compared with CTRL, which persisted for the entire postimmobilization period.

No significant effect of the factor GROUP was observed for the shoulder movements, but a significant effect of TIME emerged [*F*(2, 76) = 9.05, *P* < 0.001, partial η^2^ = 0.19, BF_10_ > 100]. However, it should be noted that AM values for the shoulder were generally higher than those for the elbow, suggesting that shoulder-joint angles were weakly impacted by the immobilization (*SI Appendix*, Fig. S1 and Table S2). This difference may have been due to the different degrees of constraint the bandage exerted on the two joints. Whereas the elbow was constrained in a fixed position, with no residual opportunity for flexion or extension, the shoulder maintained some residual mobility, which could have obscured the overall impact of immobilization on shoulder kinematics. The factorial analysis for *R*^2^ and AOff indicated that immobilization did not affect the temporal pattern of either shoulder or elbow kinematics or the absence of offset between the curves (*SI Appendix*, Table S3). The lack of significant effects on *R*^2^ and AOff excluded any biases in determining differences in AM values.

Given that the distance between the participant and the object remained constant throughout the experimental procedure, it is reasonable to ask whether the reduced elbow flexion–extension could be ascribed to a different dynamic postural adjustment of the trunk before and after immobilization. However, we ruled out this possibility by computing the displacement of the trunk during the movement and verifying the absence of any significant effect of GROUP (*SI Appendix*, Table S3).

## Discussion

In the present study, STI induced an alteration of motor performance, with participants showing longer movement duration, higher MFr, and reduced ranges of motion after immobilization. These findings are in line with previous evidence that STI impairs the motor performance of the restricted body part, even after periods of immobilization ranging from 10 to 12 h (33, 39, 40). The alterations observed in both groups appeared to be largely reversible, with an almost complete recovery of motor performance within the 10 trials administered after the immobilization (ref. 40 has similar results).

STI in healthy participants provides a neurobehavioral model for exploring the efficacy of covert motor interventions (e.g., action observation or motor imagery), which can adaptively stimulate corticomotor representations within a context of maladaptive neural plasticity, without the influence of disease-related confounding factors (36, 41–43). One could argue that the young age of our study’s participants (mean age 22.5 y) limits the generalizability of our findings to older populations. However, previous studies have shown that the activity of frontoparietal networks shared by action observation, motor imagery, and action execution (44) does not exhibit any age-dependent changes when comparing old and young populations (45), thus supporting the generalizability of our findings across age groups.

**Fig. 3.**
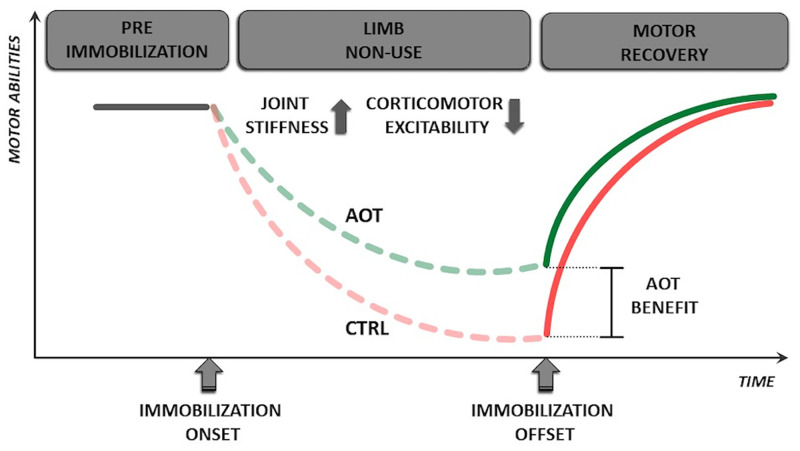
Using AOT as a tool to prevent the motor impairment caused by limb nonuse. The continuous gray line indicates a hypothetical time course of limb motor capabilities before the onset of immobilization. Motor abilities diminish at the time of injury (i.e., immobilization onset) and are represented by dashed lines. The solid red and green lines that begin after immobilization offset indicate the motor recovery of CTRL and AOT groups, respectively. “AOT benefit” indicates the advantage provided by AOT during immobilization in terms of residual motor abilities.

Most interestingly, our data show that participants receiving AOT during the immobilization period had better-preserved motor performance at the end of the immobilization period (Fig. 3). Notably, this effect pertains to the spatial organization of the movement, reflected in more increased MFr scores for the CTRL group. Conversely, the temporal features of the movement were weakly affected by the AOT intervention. This discrepancy may be due to the neural substrates of action observation, which rely heavily on frontoparietal networks encoding movement organization (1) and only to a minor extent, on the neural substrates responsible for the temporal organization of the movement (46–48).

Which neural mechanism enables AOT to prevent the decay of motor performance in healthy volunteers undergoing STI? STI is known to induce a corticomotor depression of the neural representation of the immobilized limb (41, 49, 50). Over the long term, this lowered excitability could facilitate the emergence of maladaptive behaviors or the consolidation of compensatory attitudes that, although initially beneficial for the patient, are often detrimental to the long-term outcome (51). For these reasons, the development of early-onset interventions that counteract the corticomotor depression might play a fundamental role in limiting the progressive impoverishment of motor performance.

In this regard, AOT has proven effective in limiting the STI-induced reorganization of cortical maps. Bassolino et al. (41) demonstrated that after a 10-h immobilization of the upper limb, healthy volunteers who received action observation stimuli had an almost completely preserved corticomotor map, whereas CTRL participants not receiving AOT suffered from a large reduction of the corticospinal excitability. These findings indicate that AOT has the capacity to counteract the corticomotor depression following limb nonuse, and they likely explain the neural mechanism underlying the preserved motor performance of the AOT group in our study. Given our experimental design, whether the reduced effects of immobilization were mainly driven by observing specific movements or rather, by observing any movement remains an open point. However, the notion that action observation elicits a motor activity following a somatotopic and actotopic organization (52) points at congruent actions as the ideal stimuli for an AOT. This is also in line with previous behavioral data, showing that during a physical practice task, action observation induces a strengthening of the motor memory encoding but only if the observed action is congruent with the practiced one (53).

Although our study involved healthy subjects, its results are informative for different clinical scenarios. Orthopedic and peripheral nervous system diseases (e.g., brachial plexopathy, nerve or radicular injuries, inflammatory disorders like Guillain–Barré syndrome) represent the clinical conditions closest to those of our experimental model because the brain structures hosting the mirror mechanism are intact. In all these circumstances, corticomotor depression might lead to the instantiation of dysfunctional motor behavior. AOT can promote the maintenance of a central-to-periphery interplay resembling the premorbid one, thus favoring a faster restoration of motor function.

In central nervous system disorders (e.g., stroke), the patient's inability to move is due not to peripheral constraints but rather, to the damage of brain structures responsible for the generation and control of the movement. Following the abrupt disruption of motor programs, the motor system undergoes compensatory neural processes like perilesional remapping (54, 55) and interhemispheric functional rebalancing (56, 57); thus, the view of an exclusive, progressive corticomotor depression is unsuitable here. Maladaptive neural plasticity processes might occur in this case, and AOT can still limit their instantiation. This benefit is in line with the well-known effectiveness of AOT in promoting motor recovery in poststroke patients (4, 5). However, this capacity depends largely on the lesion’s extent and topography: that is, on the postinjury functioning of the corticomotor system. A promising aspect of our findings in relation to poststroke patients is the capacity of AOT to intervene mainly in MFr, which has been described as a key feature of stroke-related motor dysfunctions (58, 59).

## Conclusions

The present study showed that administering AOT during immobilization limits the movement alterations induced by limb nonuse. Given AOT’s protective role against the decline of motor performance, action observation represents a valid, effective tool for early intervention in the motor system during limb nonuse, thus reducing the burden of further rehabilitation.

## Materials and Methods

### Participants.

Forty naïve volunteers (17 males and 23 females, mean age 22.9 ± 3.7) participated in the experiment. The sample size was calculated using a power analysis with the accepted minimum level of significance (α) of 0.05, the accepted level of power (1 − β) of 0.80, and an effect size of 0.5 (partial η^2^ = 0.2); the analysis was performed using G*Power 3.1 (60). Of the participants, 37 were right handed, and 3 were left handed [according to the Edinburgh Handedness Inventory (61)]. All the subjects reported normal or corrected-to-normal vision and no previous history of neurological disorders or recent orthopedic injuries for the dominant upper limb. The volunteers were randomly assigned to two experimental groups: 1) AOT: 9 males and 11 females, mean age 22.5 ± 2.6 and 2) CTRL: 8 males and 12 females, mean age 23.4 ± 4.6.

The local ethics committee approved the study (Comitato Etico dell' Area Vasta Emilia Nord, 10084, 12.03.2018), which was conducted according to the principles expressed in the Declaration of Helsinki. Each participant provided written informed consent before the experimental sessions.

### Immobilization Procedure.

The dominant upper limb of each participant was immobilized for a total of 16 h. The arm and forearm were wrapped with an orthopedic bandage commonly used in clinical practice. The bandage limited arm and shoulder movements by fixing the elbow joint at 90° of flexion (Fig. 1*C*). Subjects were instructed to keep the bandage on (even during the night) and to minimize upper-limb movements from the time the bandage was applied (at about 6:00 PM) to the next morning (at about 10:00 AM).

We chose a 16-h immobilization period because it would impose a nonuse period likely sufficient to evoke observable effects; indeed, previous studies have reported that significant behavioral changes in upper-limb movements following immobilization can be observed as early as 10 to 12 h after the beginning of immobilization (34, 39, 40).

Participants underwent the kinematics assessment immediately after the bandage was removed. They were instructed to maintain a relaxed position, avoiding any movement until the beginning of the motor task.

### Experimental Procedure.

Because the study evaluated the impact of AOT on preserving motor function during immobilization, the motor performance of participants was tested before (i.e., preimmobilization) and after (i.e., postimmobilization) the upper-limb nonuse period.

In the preimmobilization phase, the experimental setup required the participants to perform three actions, which required reaching toward and grasping spheres (7 cm in diameter) placed in different positions in the surrounding space. The spheres were positioned at the boundary of the peripersonal space of each participant: that is, at an arm’s-length distance, thus limiting trunk tilt during task performance.

The participants were seated comfortably on a stool while maintaining a neutral starting position, with their hands prone on their knees. They were instructed to execute the following upper-limb actions (Fig. 1*A*):

A) Reaching toward and grasping the sphere located A-Low;
B) Reaching toward and grasping the sphere located A-High;
C) Reaching toward and grasping the sphere located L-Low, compatibly with the participant's handedness.

After the execution of each movement, the participants were instructed to return to the starting position. The sequence of three movements (A–B–C) was repeated 10 times, for a total of 30 movements.

Following the kinematic assessment, the participants were immobilized as described above. Three virtual reality (VR) treatment sessions (with “actions” or “natural scenarios” stimuli) were administered for each participant. The subjects underwent the first session immediately after the immobilization and underwent the other two (with an interval of 20 min) the following morning, just before the bandage was removed. At the end of the VR sessions, the bandage was removed, and the participants immediately performed the same motor tasks used in the preimmobilization phase (Fig. 1*D*).

### VR Stimuli.

Visual stimuli were developed with immersive VR technology using a cross-platform game engine (Unity 3D; Unity Technologies) and were presented to the participants through a VR headset display (HTC Vive Pro; HTC Corporation).

Two classes of stimuli were created; the subjects in the AOT group observed reach-to-grasp tasks from a first-person egocentric perspective, whereas the participants in the CTRL group observed three different scenes depicting natural scenarios without any motor content. In the AOT scenario, a humanoid avatar performed the same sequence of movements that participants were required to perform, thus mimicking the experimental setting. The avatar was animated using the kinematics acquired from an actor.

While wearing the VR headset, subjects could rotate their head in the virtual three-dimensional (3D) environment until the movement appeared in their field of view. More precisely, subjects looked forward in the A-Low and A-High movements, whereas they rotated their heads 90° (clockwise or counterclockwise, depending on the subject’s handedness) for the L-Low movement.

Subjects observed the stimuli, each of which lasted about 1 min. In the case of actions, virtual stimuli consisted of a single movement repeated 10 times, and after observation, the participants performed a motor-imagery task: that is, they kept their eyes closed while mentally rehearsing the just-observed actions for 30 s. This 90-s block was repeated four times for each movement (3), for a total duration of about 18 min. The schedule of sessions for the CTRL group remained the same, replacing actions stimuli with natural scenarios stimuli and motor imagery with visual recall of the natural scenarios.

### Kinematic Recording and Data Analysis.

The movements performed by the participants were recorded using a marker-based 3D optoelectronic system (SMART; BTS Bioengineering), which consisted of six infrared cameras detecting the position of eight reflective spherical markers (10 mm in diameter) at a sampling rate of 120 Hz and with a spatial resolution of 0.3 mm. The markers were positioned on the subject's dominant arm following the recommendations of the International Society of Biomechanics for upper limbs (62): one marker on the suprasternal notch; one marker on the seventh cervical vertebra; one marker on the spinous process of the eight thoracic vertebra; one marker on the shoulder at the acromial edge; two markers on the elbow in the lateral and medial humerus epicondyle, respectively; and two markers on the wrist in the radius and ulnar styloid process, respectively. After reconstructing the positions of the markers, their raw trajectories underwent postprocessing (Matlab2018a; MathWorks Inc.), including low-pass filtering and the reconstruction of missing marker positions (63).

Movement analysis was focused on the reaching phase, which was segmented according to the tangential velocity of the ulnar styloid marker (64). The temporal boundaries of the reaching phase were identified as the times at which the ulnar velocity surpasses and returns below the 5% of the peak velocity (reaching start and end, respectively). Once defined the reaching phase, specific parameters were computed to evaluate the performance of the subjects (65):

• RD: the overall duration of the reaching movement (64);
• VP: the peak of the tangential velocity of the ulnar styloid marker (66);
• MFr: an index quantifying the relative ratio between the elbow and the shoulder ranges of motion used to evaluate whether the overall kinematic approach varied between pre- and postimmobilization and across groups; this index was defined following the approach reported in (67):
  $$MFr=100\text{\% }\left(1-\frac{E_{ROM}}{S_{ROM}}\right),$$
  where *E_ROM_* is the average ROM elbow flexion–extension and *S_ROM_* is the average ROM of the shoulder-joint angle (shoulder flexion–extension or abduction–adduction). A reduction of elbow ROM leads to higher MFr scores, whereas a reduction of shoulder ROM would lead to lower MFr scores.

Subsequently, shoulder-joint angles were calculated following the International Society of Biomechanics recommendations for the upper limbs (62). For the A-Low and A-High movements, shoulder flexion–extension (sagittal plane) was considered, and for the L-Low movement, shoulder abduction–adduction (coronal plane) was considered. Flexion–extension was considered to be of main importance for the elbow joint. These angles are referred to hereafter as angles of interest.

To make the temporal pattern of the movement comparable both within and across participants, all the kinematics angles were segmented and resampled to a normalized 0 to 100% time interval, thus scaling all movements to a standard duration. These data could then be compared in terms of the pattern of movement, regardless of their duration. A waveform-similarity analysis was performed using the LFM (38) separately for each angle of interest; specifically, the LFM compared each postimmobilization trial vs. the average preimmobilization performance. The LFM returns three separate indexes: 1) the goodness of the linear fit between the curves (*R*^2^), 2) the AOff, and 3) the AM index. *R*^2^ ranges from zero to one (from null to perfect similarity between the curves). The ideal AM value is one, and it indicates maximum similarity in amplitude between the curves (values lower than one indicate a shrinkage of the investigated angle; values above one indicate an increased amplitude). Given our experimental design, the AM parameter was used to test the impact of immobilization and the effect of AOT on the amplitude of angles of interest, *R*^2^ was used to reveal whether the temporal movement organization changed between pre- and postimmobilization, and AOff was used to verify the presence of measurement errors. The last two indices were used as control variables to test the reliability of AM results, excluding any biasing effect due to curve shifting (68).

Moreover, although the objects to be reached toward and grasped were systematically located within the subject’s peripersonal space, thus not requiring any tilt of the trunk during the movement, we verified the absence of compensatory trunk movements during reaching by computing the suprasternal notch marker’s maximal displacement from its initial position.

To summarize, we obtained 10 values of performance parameters and LFM coefficients for each movement and subject. The within-subjects difference relative to baseline was computed for RD, VP, and MFr. These variables expressed postimmobilization trial-by-trial variation of motor performance across time (T1–T2–T3–T4–T5–T6–T7–T8–T9–T10) relative to the average baseline values (T0).

### Statistical Analysis.

A series of unpaired *t* tests was conducted between groups on the preimmobilization scores of each kinematic parameter to rule out the possibility of any significant difference between AOT and CTRL at baseline (*SI Appendix*, Table S1).

A mixed-design ANOVA was carried out on the main parameters (RD, VP, MFr, AM_Elb_, AM_Sho_) with MOVEMENT (A-Low, A-High, L-Low) and TIME as within-subjects factors and GROUP (i.e., AOT/CTRL) as a between-subjects factor. The same statistical design was applied to the control parameters (*R*^2^_Elb_, AOff_Elb_, *R*^2^_Sho_, AOff_Sho_, Trunk) to rule out the possibility of any significant effect of TIME and GROUP factors.

Previous literature has shown that functional recovery in rehabilitation typically follows a logarithmic trend (69–71). We leveraged this principle to reduce the number of within-subjects levels. That is, we identified the trials presumed to express 50 and 95% of the recovery: T4 and T9, respectively. Consequently, the ANOVA was carried out with the trials at T1, T4, and T9 as within-subjects factors.

Considering the number of ANOVAs, we applied the Šidák–Bonferroni correction to the main analyses, setting the significance threshold at *P* = 0.01 (0.05 divided by five variables). Post hoc tests were performed using the Newman–Keuls correction for multiple comparisons. All variables were normally distributed as verified by the Kolmogorov–Smirnov test (*P* > 0.05). Partial η^2^ was calculated as a measure of effect size.

Bayesian statistics were implemented to measure the probability of the real influence of the tested factors on movement kinematics. The BF was computed to express how many times the alternative hypothesis (H1) is more likely to occur than the null hypothesis (H0) for the main variables (BF_10_) or how many times H0 is more likely to occur than H1 for the control variables (BF_01_). The BF robustness check (*SI Appendix*, Table S2) indicates the level of evidence (72). 

## Supplementary Material

Supplementary File

## Data Availability

The raw data used in the present study are available at Zenodo at the following link: https://doi.org/10.5281/zenodo.5603250.
