# Supplementary material for: Observation of others’ actions during limb immobilization prevents the subsequent decay of motor performance
Source: Proc Natl Acad Sci U S A. 2021 Nov 15;118(47):e2025979118. doi: 10.1073/pnas.2025979118 (PMC8617512; doi:10.1073/pnas.2025979118)
Supplement: Supplementary File [file pnas.2025979118.sapp.pdf]

PNAS
www.pnas.org

Supplementary Information for

Observation of others' actions during limb immobilization prevents the subsequent decay of motor performance

Doriana De Marco[a,§,*], Emilia Scalona [a,§], Maria Chiara Bazzini[a,b], Arturo Nuara [a], Elisa Taglione[c], Nicola Francesco Lopomo[a,d], Giacomo Rizzolatti[a,b,*], Maddalena Fabbri-Destro [a,°], Pietro Avanzini[a,e,°]

a) Consiglio Nazionale delle Ricerche, Istituto di Neuroscienze, Parma, Italy
b) Università degli Studi di Parma, Dipartimento di Medicina e Chirurgia, Italy
c) INAIL, Centro di Riabilitazione Motoria di Volterra, Italy
d) Università degli Studi di Brescia, Dipartimento di Ingegneria dell'Informazione, Italy
e) Istituto Clinico Humanitas, Milano, Italy

§These authors contributed equally to the study.
° These authors contributed equally to the study.

Corresponding authors: Giacomo Rizzolatti and Doriana De Marco
Email: giacomo.rizzolatti@unipr.it; doriana.demarco@in.cnr.it;

This PDF file includes:

Figures S1
Tables S1 to S3

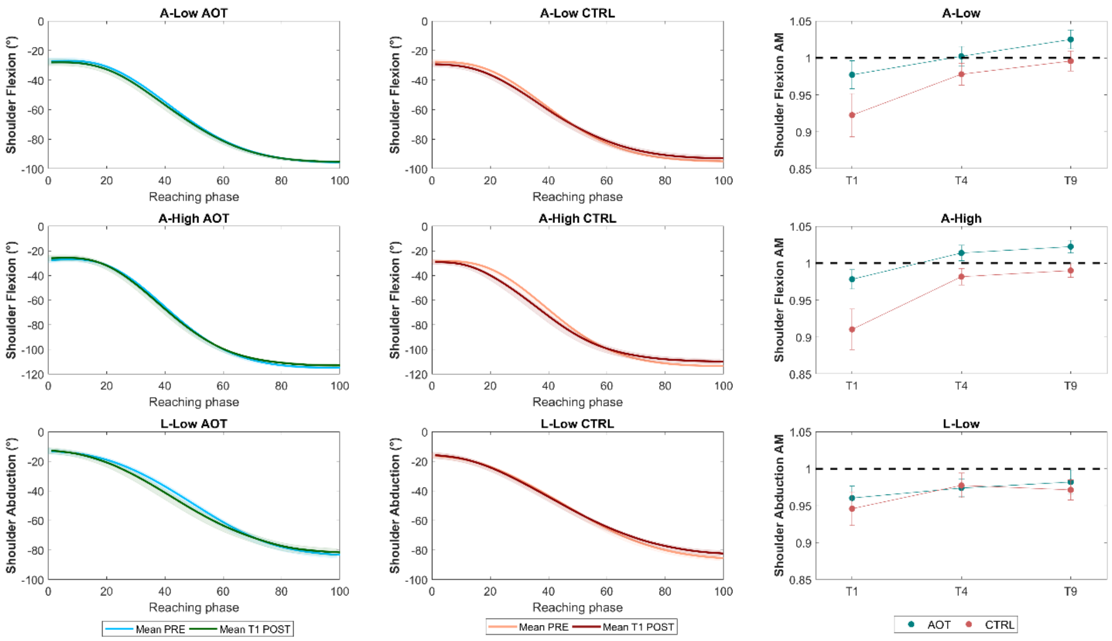

Fig. S1. Left and center columns: time courses of shoulder angles averaged across preimmobilization trials (light colors) for the AOT and CTRL groups (green and red, respectively) for the three movements (A-Low, A-High, and L-Low); time courses of mean shoulder angles acquired during the first trial of the postimmobilization phase (T1) are superimposed in corresponding dark color tones. Right column: means and standard errors of amplitude modulation (AM) evaluated at T1, T4, and T9 for the AOT and CTRL groups (green and red, respectively) and the three movements (A-Low, A-High, and L-Low).

Mov	Index	AOT baseline	CTRL baseline	*p*-value
A-Low	RD (s)	0.96 (0.11)	1.03 (0.14)	0.18
	VP (m/s)	1.32 (0.21)	1.30 (0.21)	0.71
	MFr (%)	34.12 (20.31)	38.91 (13.36)	0.42
A-High	RD (s)	1.04 (0.13)	1.05 (0.14)	0.79
	VP (m/s)	1.60 (0.25)	1.57 (0.25)	0.78
	MFr (%)	37.39 (14.77)	40.72 (12.00)	0.44
L-Low	RD (s)	1.19 (0.26)	1.22 (0.20)	0.70
	VP (m/s)	1.36 (0.23)	1.37 (0.23)	0.96
	MFr (%)	28.63 (24.89)	27.30 (23.66)	0.86

Table S1. Means and standard deviations of the preimmobilization scores (baseline) of reaching duration (RD), velocity peak (VP), and movement fractionation (MFr) for both groups (AOT and CTRL) and each movement (A-Low, A-High, and L-Low). The *p*-values are relative to unpaired *t*-tests between groups.

		ANOVA				Bayesian Analysis	
**Movement**	**Index**	**F**	***p***	**η^2**	**post-hoc**	**BF_{10}**	**BF Robustness**
**Main Effect Time**	**RD (s)**	18.83	<0.001	0.33	T1 > T4<br>T1 > T9	> 100	Extreme
	**VP (m/s)**	28.27	<0.001	0.42	T1 < T4<br>T1 < T9	> 100	Extreme
	**MFr (%)**	10.41	<0.001	0.21	T1 > T4<br>T1 > T9	> 100	Extreme
	**AM_{Elb}**	10.06	<0.001	0.21	T1 < T4<br>T1 < T9	> 100	Extreme
	**AM_{Sho}**	9.05	<0.001	0.19	T1 < T4<br>T1 < T9	> 100	Extreme
**Main Effect Movement**	**RD (s)**	1.78	0.175	0.04		0.12	No Evidence
	**VP (m/s)**	2.36	0.101	0.06		0.32	No Evidence
	**MFr (%)**	9.90	<0.001	0.21	L-Low>A-Low<br>L-Low>A-High	> 100	Extreme
	**AM_{Elb}**	8.69	<0.001	0.22	L-Low<A-Low<br>L-Low<A-High	48.91	Very Strong
	**AM_{Sho}**	1.22	0.299	0.03		0.11	No Evidence
**Main Effect Group**	**RD (s)**	0.41	0.524	0.01		0.27	No Evidence
	**VP (m/s)**	0.36	0.554	0.01		0.19	No Evidence
	**MFr (%)**	7.55	0.009	0.16		5.15	Moderate
	**AM_{Elb}**	10.98	0.002	0.22		15.94	Strong
	**AM_{Sho}**	3.76	0.06	0.09		1.01	No Evidence

Table S2. ANOVA and Bayesian results for the main variables: reaching duration (RD), velocity peak (VP), movement fractionation (MFr), amplitude modulation of the elbow (AM_{Elb}), and amplitude modulation of the shoulder (AM_{Sho}).

		ANOVA				Bayesian Analysis	
**Movement**	**Index**	**F**	**p**	**η^2**	**post-hoc**	**BF_{01}**	**BF Robustness**
**Main Effect Time**	**R^2_{Elb}**	2.79	0.067	0.07		0.84	No Evidence
	**$AOff_{Elb}$**	0.06	0.943	0.00		30.25	Very Strong
	**R^2_{Sho}**	7.30	0.001	0.16	T1 < T4<br>T1 < T9	0.04	No Evidence
	**$AOff_{Sho}$**	0.50	0.605	0.01		12.72	Strong
	**Trunk (mm)**	0.14	0.866	0.04		29.39	Strong
**Main Effect Movement**	**R^2_{Elb}**	10.36	<0.001	0.21	L-Low<A-Low<br>L-Low<A-High	< 0.01	No Evidence
	**$AOff_{Elb}$**	5.25	0.007	0.12	L-Low<A-Low<br>L-Low<A-High	0.08	No Evidence
	**R^2_{Sho}**	2.54	0.085	0.06		5.77	Moderate
	**$AOff_{Sho}$**	0.33	0.717	0.01		22.93	Strong
	**Trunk (mm)**	2.70	0.073	0.06		0.25	No Evidence
**Main Effect Group**	**R^2_{Elb}**	0.32	0.573	0.01		3.85	Moderate
	**$AOff_{Elb}$**	0.55	0.461	0.01		2.49	Anecdotal
	**R^2_{Sho}**	1.01	0.321	0.03		4.05	Moderate
	**$AOff_{Sho}$**	1.49	0.229	0.03		2.62	Anecdotal
	**Trunk (mm)**	1.66	0.205	0.04		0.51	No Evidence

Table S3. ANOVA and Bayesian results for the control variables: R^2 of the elbow (R^2_{Elb}), offset of the elbow ($AOff_{Elb}$), R^2 of the shoulder (R^2_{Sho}), offset of the shoulder ($AOff_{Sho}$), and trunk compensation movement (Trunk).
